# Transfer of tactile perceptual learning to untrained neighboring fingers reflects natural use relationships

**DOI:** 10.1152/jn.00181.2015

**Published:** 2015-12-02

**Authors:** Harriet Dempsey-Jones, Vanessa Harrar, Jonathan Oliver, Heidi Johansen-Berg, Charles Spence, Tamar R. Makin

**Affiliations:** ^1^FMRIB Centre, Nuffield Department of Clinical Neurosciences, University of Oxford, Oxford, United Kingdom;; ^2^School of Psychology, University of Queensland, Brisbane, Australia;; ^3^School of Optometry, University of Montreal, Montreal, Quebec, Canada; and; ^4^Department of Experimental Psychology, University of Oxford, Oxford, United Kingdom

**Keywords:** perceptual learning, primary somatosensory cortex, co-activation, generalization, topography

## Abstract

Tactile learning transfers from trained to untrained fingers in a pattern that reflects overlap between the representations of fingers in the somatosensory system (e.g., neurons with multifinger receptive fields). While physical proximity on the body is known to determine the topography of somatosensory representations, tactile coactivation is also an established organizing principle of somatosensory topography. In this study we investigated whether tactile coactivation, induced by habitual inter-finger cooperative use (use pattern), shapes inter-finger overlap. To this end, we used psychophysics to compare the transfer of tactile learning from the middle finger to its adjacent fingers. This allowed us to compare transfer to two fingers that are both physically and cortically adjacent to the middle finger but have differing use patterns. Specifically, the middle finger is used more frequently with the ring than with the index finger. We predicted this should lead to greater representational overlap between the former than the latter pair. Furthermore, this difference in overlap should be reflected in differential learning transfer from the middle to index vs. ring fingers. Subsequently, we predicted temporary learning-related changes in the middle finger's representation (e.g., cortical magnification) would cause transient interference in perceptual thresholds of the ring, but not the index, finger. Supporting this, longitudinal analysis revealed a divergence where learning transfer was fast to the index finger but relatively delayed to the ring finger. Our results support the theory that tactile coactivation patterns between digits affect their topographic relationships. Our findings emphasize how action shapes perception and somatosensory organization.

historically, invaluable insights regarding the neural architecture of the somatosensory system and primary somatosensory cortex (SI) have been achieved using electrophysiological studies in nonhuman primates. Because of these efforts, we now have a fairly fine-grained understanding of individualization vs. overlap of finger representations resulting from single- or multidigit receptive fields (RFs), respectively (hereafter simply “finger overlap”). However, interspecies differences in finger movements are well documented (Schieber 1991; reviewed in Häger-Ross and Schieber 2000). Given the bidirectional relationship between the somatosensory and motor systems (Darainy et al. 2013), it is likely there are many fundamental differences in the patterns of finger overlap between humans and nonhuman primates. Consequently, the precise nature of inter-finger relationships in the human somatosensory system remains to be effectively explored. Unfortunately, the invasive nature of electrophysiological recordings and the resolution of available neuroimaging techniques limit the investigation of RF properties in humans. Subsequently, alternative methods of revealing inter-finger relationships in the somatosensory system of humans are required. In the current study, we used a tactile perceptual learning paradigm as a tool to probe somatosensory finger overlap.

Perceptual learning is the inherent ability of sensory systems to improve following repeated exposure to stimuli (Gibson 1969). Tactile perceptual learning (Volkman 1858) has been shown to transfer from trained to untrained fingers that overlap in their somatosensory finger representations (with no transfer to nonoverlapping fingers: Harrar et al. 2014; similar results in Harris et al. 2001). In the present study, we used tactile perceptual learning to investigate the relationship between fingers in the human somatosensory system.

Cortical maps in the somatosensory system are thought to emerge through input-dependent (Hebbian) synaptic changes, as shown in electrophysiological research (Recanzone et al. 1992a; Wang et al. 1995). This is reflected in the organization of the SI, where adjacent fingers are represented next to each other on the cortical surface (Kaas et al. 1979). It has been suggested that this organization occurs because temporal schedules of input are more similar for body parts that are physically close compared with body parts that are further away (as modeled by Detorakis and Rougier 2014). The impact of temporally (a)synchronous input on cortical organization has been demonstrated experimentally through surgical attachment of fingers (syndactyly: Clark et al. 1988; see also Allard et al. 1991), repetitive co-stimulation across adjacent fingers (Wang et al. 1995) or single fingers (Jenkins et al. 1990; Recanzone et al. 1992a, 1992b), and following highly stereotypic movements with subsequent repetitive tactile inputs (Byl et al. 1996; Sterr et al. 1998). Converging evidence from these studies suggests synchrony of tactile inputs causes “fusing” of co-stimulated finger representations such that the strict boundaries between the fingers become blurred (and vice versa for asynchronous inputs).

The fingers engage in highly regular patterns of joint angular covariances and muscular co-contraction patterns. These patterns are position and muscular “synergies” (Santello et al. 1998; Weiss and Flanders 2004; see Lederman and Klatzky 1993 for similar results during haptic exploration). In humans, these patterns of co-use (Reilly and Hammond 2000; Zatsiorsky et al. 1998) have been tracked in natural settings (Belić and Faisal 2015; Ingram et al. 2008) and laboratory settings (Häger-Ross and Schieber 2000; Soechting and Flanders 1997). It has been found that during hand actions, fingers cooperate to varying degrees: the middle and ring fingers, for example, operate more frequently together than the index and middle fingers, whereas the index finger engages in more independent use than the middle or ring finger (Belić and Faisal 2015; Ingram et al. 2008). These synergies, which result from musculoskeletal as well as neural constraints (Lang and Schieber 2004; Reilly and Schieber 2003; Soechting and Flanders 1997), simplify motor control by reducing the degrees of freedom of the hand (Tresch et al. 2006).

Recent evidence suggests that patterns of use can also affect the organization of cortical representations. For example, Overduin et al. (2012, 2014) demonstrated a relationship between hand- and arm-muscle synergies and motor cortex organization using microstimulation in monkeys. In humans, Ejaz et al. (2015) reported that multivoxel patterns of representational similarity in sensorimotor cortex are predicted by the statistics of natural finger movements. Together, these findings suggest that everyday activities that involve the hand may shape the underlying organization of the somatosensory cortex by causing distinct tactile coactivation patterns (see discussion in Flanders 2005).

In the current study we predicted that distinct use patterns, leading to different extents of overlap between finger representation zones, would result in a dissociation in the ability of the fingers to learn from transfer of perceptual learning (also known as the “generalization” of perceptual learning). We recently demonstrated that after 4 days of tactile training, learning transfers from a trained finger to adjacent and homologous fingers, but not to other fingers (Harrar et al. 2014). That is, learning transferred to fingers with overlapping somatosensory representations. In the current study, we investigated the transfer of tactile learning from the middle finger to the index and ring fingers. This allowed us to compare two fingers with the same neighboring relationship to the trained finger (i.e., cortically and physically “adjacent”) but of differing use patterns with respect to the trained finger. Although both the adjacent index and adjacent ring fingers should receive significant learning gains from the trained middle finger by posttest, we expected the extent of cortical overlap of these fingers with the trained finger would cause a different pattern of learning through transfer.

One mechanism by which cortical overlap could affect transfer of leaning is cortical magnification. Intensive tactile stimulation has been shown to result in an increased representation of the trained skin surface, as demonstrated both by experimental training (Byl et al. 1996, 1997; Jenkins et al. 1990; Recanzone et al. 1992a, 1992b) and natural exposure (Sterr et al. 1998; Xerri et al. 1994), and through computational models (Detorakis and Rougier 2014). It follows then that the increase in the cortical representation of the trained middle finger could occur at the expense of its neighboring representations, i.e., subsuming of cortical resources (although see discussion for alternative mechanisms of learning patterns). Since the middle finger is predicted to overlap more with the ring finger (than the index finger), we predict cortical magnification of the middle finger to cause more interference in the ring than the index finger. Over time, temporary learning-induced cortical changes subside (Reed et al. 2011). We therefore predict that training the middle finger will initially hinder performance in the ring finger, but eventually, when benefits from learning outweigh the interference, there will be significant perceptual gains in both the index and ring fingers adjacent to the trained finger.

## METHODS

### 

#### Participants.

Twenty-six individuals were randomly assigned to the trained and control groups. Partial data from five individuals were discarded due to malfunctions during data collection, leaving *n* = 12 in the trained group (mean age 28 yr; 7 females) and *n* = 9 in the control group (mean age 25 yr; 6 females). All participants gave their informed consent, and ethical approval for the study was granted from the medical sciences interdivisional research ethics committee of the University of Oxford (Reference: MSD-IDREC-C1-2013-102).

#### General procedure.

**Fig. 1.**
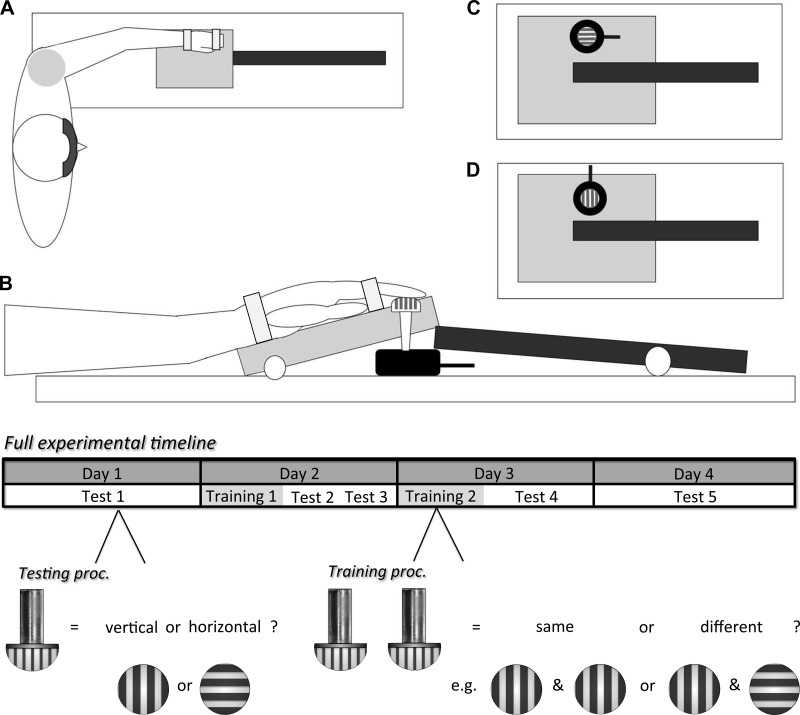
*Top*: schematic of the stimulus presentation apparatus. *A*: participants were blindfolded and their hand positioned prone on a wooden support and secured with Velcro straps. *B*: cross section of finger contacting tactile grating, which protruded through the aperture underneath the distal pad of the finger in the hand support. *C* and *D*: grating in the rotating disk at vertical (*C*) and horizontal (*D*) orientations. The apparatus and disk were controlled by the experimenter. *Bottom*: full experimental timeline for training and testing across the 4 days of the protocol. Participants were presented with gratings of varying groove width (0.25–3.5 mm) in a semirandom order. During testing sessions, participants determined the orientation of an individual grating (vertical or horizontal). During training sessions, participants determined whether 2 grating presented consecutively were the same or not in orientation. Feedback on performance was provided after every block (during testing sessions) or every trial (during training sessions).

The procedure was conducted over 4 days. The testing and training procedures were adapted from Harrar et al. (2014). Participants in the experimental group received a protocol of two training sessions, interspersed with five testing sessions (see timeline in Fig. 1). The untrained (control) group followed an identical sequence of five testing sessions but did not receive any training (i.e., same exposure to testing procedures but no direct training). Participants were blindfolded for the duration of all sessions.

#### Testing procedure.

Testing sessions were used to determine acuity in tactile grating orientation (Sathian and Zangaladze 1996; Van Boven and Johnson 1994). This measure is a highly robust and reliable indicator of tactile acuity and overcomes various pitfalls of other measures of tactile acuity, such as two-point discrimination (Bleyenheuft and Thonnard 2007; Johnson and Phillips 1981; Sathian and Zangaladze 1996; Van Boven and Johnson 1994; see Vega-Bermudez and Johnson 2002 for further critique). Seven plastic dome gratings varying in groove width and isometric groove spacing were selected for testing (Stoelting, Wood Dale, IL). The selected spacings were 0.25, 0.5, 1.0, 1.2, 1.5, 2.5, and 3.5 mm. The glabrous surface of the distal pad of the left and right index, middle, and ring fingers were tested. Gratings were presented using a semiautomated lever device operated by an experienced experimenter (as in Harrar et al. 2014): the participant's hand rested on a wooden block (see Fig. 1). The finger selected for testing was positioned over a small hole under which the selected grating was placed, facing upward. Depression of the lever by the experimenter tilted the participant's hand slightly upward, allowing the grating underneath to be oriented either vertically or horizontally (i.e., parallel or orthogonal to the finger, respectively). The lever was then released, and gravity allowed the hand to drop, causing contact between the grating and finger (through the hole, which was 1 cm in diameter, just smaller than the grating diameter). Contact lasted for ∼1 s, with an approximate interstimulus-interval (ISI) of 2–3 s, depending on the speed of the participant's response on that trial.

Participants reported the perceived orientation by selecting the left or right buttons of a mouse (2-alternative forced choice; 2AFC). Each grating was presented for 1 block of 10 trials on each finger (5 vertical, 5 horizontal). To increase the number of trials for gratings close to the individual's perceptual threshold, four gratings that were within the dynamic accuracy range of each finger were selected for an additional block of presentation. That is, gratings were not re-presented if 90–100% accuracy was achieved the first time they were tested. When <90% accuracy was achieved for all gratings, the four maximally sized gratings were selected for re-presentation. Grating orientation (on each trial) and grating width (blocks) were organized in a pseudorandom order, controlled by a computer (MATLAB release 2013a; The MathWorks, Natick, MA). After each block, accuracy feedback was given over headphones (0–100% for that block). Participants were instructed to prioritize accuracy over speed, and no time limit was imposed for responses. Each session lasted ∼1 h (with short interblock breaks).

#### Training procedure.

For the trained group only, training was conducted on the middle finger of the right or left hand (6 in each subgroup). Four gratings were selected for training from 11 gratings that ranged in width (0.25, 0.3, 0.5, 0.75, 1.0, 1.2, 1.5, 2.0, 2.5, 3.0, or 3.5 mm). The four were selected so that two gratings were above the participant's tactile threshold (as measured from data in the preceding testing block; see below for threshold calculation procedure), i.e., larger in width, and two selected below threshold i.e., smaller. This threshold-based selection was used to provide stimuli of a sufficient difficulty level to allow optimal learning transfer (Ahissar and Hochstein 1997). If the participant's threshold was too close to the maximum grating size, selection above and below the threshold was not possible. In this case the largest four gratings were used.

The participants' task during training was to determine whether two consecutive gratings had the same or a different orientation (2AFC; see Fig. 1). The training task was a modification of the testing task to encourage learning of orientation rather than specific task requirements. Accuracy in orientation detection was provided over headphones (“correct”/“incorrect”) after every trial to maximize learning. Accuracy was encouraged over speed and no time limit imposed. Training sessions lasted ∼50 min, including short interblock breaks.

#### Determining perceptual thresholds.

Accuracy in orientation discrimination during the testing procedure was plotted as a function of grating size. Data were fitted with a three-parameter Weibull psychometric function using the Palamedes toolbox in MATLAB (release 1.6.0; Prins and Kingdom 2009; http://www.palamedestoolbox.org). The threshold was interpolated from the grating size estimated to yield 82% accuracy. Overall, the Weibull function produced a good mean fit (pDev; *M* = 0.70). For a small number of data, the function did not converge (6.5% of the total data set). These missing thresholds were replaced with the mean of the thresholds from the previous and the subsequent session, for that participant and finger. These thresholds were used for all statistical analyses presented below.

#### Statistical analysis.

Between- and within-participants comparisons were assessed using mixed-model analysis of variance (ANOVA). When appropriate, contrasts analysis was used to determine if a significant pattern existed in the data set (e.g., linear improvement in thresholds over time). Planned comparisons, strictly used to replicate previous findings, were assessed using two-tailed paired-sample *t*-tests (effect size reported using Cohen's *d*). To more appropriately represent within-participant variance, the error bars plotted in the figures were calculated using procedures described in Cousineau (2005) and corrected using the adjustment described in Morey (2008). To provide additional information regarding the pattern of learning over time in the trained group, we also analyzed the data with the generalized estimating equation (GEE) technique.

## RESULTS

### 

#### Baseline verification.

To ensure baseline consistency within our sample, finger thresholds at baseline were compared across groups and hands. A mixed ANOVA with within-participants factors Finger (3 levels: index, middle, and ring) and Hand (2 levels: left and right) and the between-participants factor Group (2 levels: trained and control) was tested. There was no main effect of Group (*P* = 0.827) or interaction of Group with the other factors (0.526 < *P* > 0.987), confirming no differences between groups at baseline. A main effect of Finger was found [*F*(2,38) = 10.51, *P* < .001, η_p_^2^ = 0.36], where the index finger had the lowest threshold at baseline, followed by the middle and then ring fingers, consistent with previous research (Harrar et al. 2014; Vega-Bermudez and Johnson 2001; see Table 1 for additional statistics).

**Table 1.** Details of main effects and interactions for finger thresholds at baseline

Factor	Are baseline thresholds equivalent across trained and control groups?
Finger	*F*(2,38) = 10.51
	***P* < 0.001** (η^2^_p_ = 0.36)
Hand	*F*(1,19) = 0.42
	*P* = 0.526 (η^2^_p_ = 0.02)
Group	*F*(1,19) = 0.05
	*P* = 0.827 (η^2^_p_ = 0.01)
Finger × group	*F*(2,38) = 0.01
	*P* = 0.987 (η^2^_p_ = 0.01)
Hand × group	*F*(1,19) = 0.15
	*P* = 0.701 (η^2^_p_ = 0.01)
Finger × hand	*F*(2,38) = 0.20
	*P* = 0.817 (η^2^_p_ = 0.01)
Finger × hand × group	*F*(2,38) = 0.16
	*P* = 0.855 (η^2^_p_ = 0.01)

Values are main effects and interactions not included in the text (see *Baseline verification*). Significance at *P* < 0.05 is indicated by bold text.

#### Topographic transfer patterns in the trained but not the control group.

In the control group, we predicted a small amount of learning across all fingers due to their repeated exposure to tactile stimuli during the five testing sessions (for review see Seitz and Dinse 2007). In the control group, the six fingers tested were equally exposed to the testing stimulus; thus learning was anticipated to be consistent across all fingers. This result would present itself as a main effect of Session but a nonsignificant interaction of Finger × Session.

In contrast, in the trained group, although the six fingers were equally tested, one finger received additional stimulation during the two training sessions. Thus in the trained group we anticipated topographic transfer of learning; i.e., the trained, adjacent, and homologous fingers would learn, but other fingers would not (Harrar et al. 2014). This uneven transfer of learning across fingers would be demonstrated by a significant interaction of Finger × Session for the trained group.

**Fig. 2.**
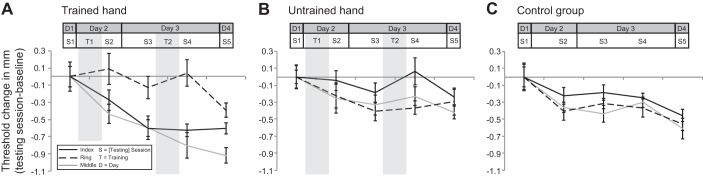
Tactile discrimination thresholds across testing sessions. Group mean thresholds (±within-participants SE) across 5 testing sessions (S) before, between, and after training (T) on the index finger (black solid line), ring finger (black dashed line), and middle fingers (gray solid line). Lower threshold values indicate greater tactile sensitivity. Values have been baseline-normalized for ease of visual comparison. Thresholds are presented for the trained group, trained hand: adjacent index, adjacent ring, and trained middle finger (*A*), the trained group, untrained hand: other index, other ring, and homologous middle finger (*B*), and control group (no training): index, ring, and middle finger means (right/left collapsed; *C*). Whereas on the trained hand tactile learning diverged between the index and ring fingers, perceptual learning progressed evenly for all fingers on the untrained hand and in the control group.

To dissociate between these training- and testing-based improvements, a mixed ANOVA was conducted with two within-participant factors: Finger (6 levels: left or right index, middle, and ring) and Session (5 levels: *sessions 1–5*), and one between-participants factor: Group (2 levels: trained and control). This analysis revealed a significant three-way interaction [*F*(9,178) = 1.65, *P* = 0.039, η_p_^2^ = 0.076; see Table 2 for other lower order significant effects]. This interaction indicated that, as predicted, learning occurred differently over fingers and testing sessions between the two groups (see Fig. 2, *A* vs. *C*). To test the distinct hypotheses laid out above for trained and control groups, we followed up this three-way interaction by testing the Finger × Session interactions separately for the trained and control groups.

**Table 2.** Details of main effects and interactions of Finger, Session, and Condition in trained and control groups

Factors	Finger (6) × Session (5) × Group (2): Trained vs. Control Group	Finger (6) × Session (5): Trained Group Only	Finger (6) × Session (5): Control Group Only
Finger	*F*(1,19) = 37.85	*F*(3,27) = 11.56	*F*(5,40) = 3.94
	***P* < 0.001** (η^2^_p_ = 0.67)	***P* < 0.001** (η^2^_p_ = 0.51)	***P* = 0.005** (η^2^_p_ = 0.33)
Session	*F*(1,19) = 39.67	*F*(4,44) = 13.13	*F*(2,14) = 10.12
	***P* < 0.001** (η^2^_p_ = 0.68)	***P* < 0.001** (η^2^_p_ = 0.54)	***P* = 0.003** (η^2^_p_ = 0.56)
Condition	*F*(1,19) = 0.02		
	*P* = 0.939 (η^2^_p_ = 0.00)		
Finger × Condition	*F*(1,19) = 1.17		
	*P* = 0.294 (η^2^_p_ = 0.06)		
Session × Condition	*F*(1,19) = 4.75		
	***P* = 0.042** (η^2^_p_ = 0.20)		
Finger × Session	*F*(1,19) = 5.76	*F*(6,69) = 1.93	*F*(5,41) = 0.76
	***P* = 0.027** (η^2^_p_ = 0.23)	***P* = 0.012** (η^2^_p_ = 0.15)	*P* = 0.758 (η^2^_p_ = 0.09)
Finger × Session × Condition	*F*(1,19) = 4.60		
	***P* = 0.045** (η^2^_p_ = 0.12)		

Values are main effects and interactions not included in the text (see results). Significance at *P* < 0.05 is indicated by bold text.

For the control group, as predicted, there was a significant main effect of testing Session [*F*(2,14) = 10.12, *P* < 0.001, η_p_^2^ = 0.56] but no interaction of Finger × Session (*P* = 0.586; see Fig. 2*C* and Table 2). The consistency across fingers supports our previous findings that fingers with different initial thresholds do not have different capacities for learning due to exposure to testing (Harrar et al. 2014).

**Fig. 3.**
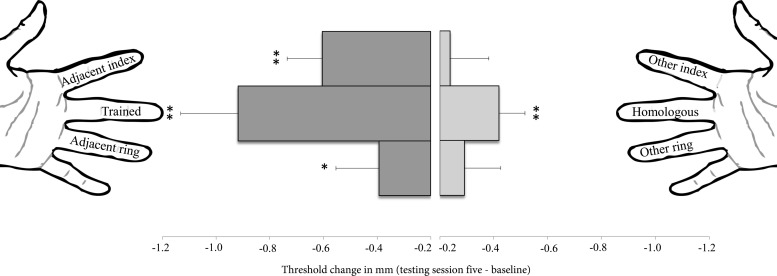
Topographic tactile perceptual learning effects in the trained group. Bars depict group mean difference between baseline (*session 1*) and posttest (*session 5*) for each of the 6 fingers tested (+within-participants SE of that difference). Larger negative values (i.e., larger bars) reflect greater improvement in tactile acuity. Paired-sample *t*-tests revealed selective reductions (significant learning gains) in the tactile threshold of the trained, adjacent index, adjacent ring, and homologous fingers consequential to training, consistent with topographic finger interrelationships in the somatosensory cortex. *P < 0.05; **P ≤ 0.001.

For the trained group, the six fingers were compared according to their relationship to the trained finger: trained, adjacent index, adjacent ring, homologous, other index, and other ring (see finger labeling in Fig. 3). As predicted, the repeated-measures ANOVA revealed a significant interaction of Finger × Session [*F*(20,220) = 1.93, *P* = 0.012, η_p_^2^ = 0.15]. This indicates that learning occurred differently across fingers throughout the experiment (see Table 2 for other statistical effects).

Subsequent comparisons between the baseline (*session 1*) and posttest (*session 5*) for each of the fingers revealed a significant improvement for the trained finger [*t*(11) = 4.63, *P* = 0.001, *d* = 1.24] and topographically related fingers [adjacent index: *t*(11) = 4.30, *P* < 0.001, *d* = 1.34; adjacent ring: *t*(11) = 2.46, *P* = 0.032, *d* = 0.71; homologous: *t*(11) = 4.40, *P* = 0.001, *d* = 1.27]. The change in the other fingers did not reach significance [although a trend toward improvement was evident: other index: *t*(11) = 1.77, *P* = 0.105; other ring: *t*(11) = 2.03, *P* = 0.067; see Fig. 3].

#### Linear change in thresholds for trained and adjacent index fingers, but not the adjacent ring or remaining fingers.

We next used contrast analysis to investigate the function that best fits the pattern of learning over time. We wished to see if these functions were different for the six trained fingers. There was a significant linear effect for the Finger × Session interaction [*F*(1,11) = 8.23, *P* = 0.015, η_p_^2^ = 0.43], whereas higher order contrasts (e.g., second and third order) did not reach significance. This significant linear interaction effect indicates a significant difference in the slopes of learning curves fit to the thresholds of each finger. Therefore, there was a difference in pattern of threshold change over time between the six trained fingers (Fig. 2, *A* and *B*).

To pinpoint the differences underlying this significant interaction, a GEE analysis was used to model the effects of Finger and Session on tactile thresholds (see *Eq. 1* in Table 3 legend for the general form of the model). The model was constrained to linear fits and was used to independently predict thresholds in all six fingers of the trained group at each testing session. The trained finger was the non-unique (redundant) parameter in the model; i.e., improvement in the trained finger was represented by the slope (β) of the main effect of Session. The model was a good fit to the data [quasi-likelihood under the independence model criterion (QIC) = 151; see Table 3 for β and *P* values for each variable]. Replicating the outcome of the mixed-model ANOVA analysis above, Finger (Wald χ^2^ = 31.9, *P* < 0.001), Session (Wald χ^2^ = 40.8, *P* < 0.001), and the Finger × Session interaction (Wald χ^2^ = 36.1, *P* < 0.001) were significant predictors of tactile orientation discrimination.

**Table 3.** Output from GEE analysis of trained group (trained hand) and presentation of modeling equations

Predictors	β Value	*P* Value
Intercept	2.26	*P* < 0.001
Adjacent index	−0.38	*P* = 0.076
Adjacent ring	−0.03	*P* = 0.830
Other ring	−0.02	*P* = 0.930
Homologous	−0.38	*P* = 0.051
Other index	−0.71	*P* = 0.001
Session (linear)	−0.22	*P* < 0.001
Adjacent index × Session	0.06	*P* = 0.249
Adjacent ring × Session	0.14	***P* < 0.001**
Other ring × Session	0.15	***P* = 0.035**
Homologous × Session	0.14	***P* = 0.022**
Other index × Session	0.18	***P* = 0.004**

Values from generalized estimating equation (GEE) analysis present factor (Finger) and covariates (Session) significantly affecting tactile acuity thresholds (β and *P* values). Session was treated as a continuous covariate variable, whereas Finger was treated as a categorical variable. The trained finger was the control (when the other factors are zero) against which the other fingers were compared. Thus a significant β value (significance at *P* < 0.05 indicated by bold text) indicates a diffference in the time course of learning compared with the trained finger. Since an increase in threshold indicates a decline in performance, the more positive is the slope, the larger the decline (i.e., less learning than the trained finger). *Equation 1*: general form Threshold_general_ = Intercept + Ai + Ar + Or + H + Oi + Session + Ai(Session) + Ar(Session) + Or(Session) + H(Session) + Oi(Session) *Equation 2*: only significant predictors Threshold = 2.26 − 0.71(oi) − 0.22(Session) + 0.14(Ar)(Session) + 0.15 (Or)(Session) + 0.14(H)(Session) + 0.18(Oi)(Session).

The β values representing the interactions for the untrained fingers were significant in all cases except for the adjacent index finger (Table 3). This supports divergent learning in the adjacent index and adjacent ring fingers. The adjacent index finger followed the same linear time course of learning as the trained finger. In contrast, the adjacent ring finger (and homologous, other index, and other ring) followed a different, nonlinear time course.

#### Time course of learning transfer.

We devised a “time-to-learn” analysis to determine how long it took for each finger to significantly improve acuity threshold with respect to baseline. Paired-sample *t*-tests were used to compare the baseline threshold (testing *session 1*) with the next time point. If this difference was not significant, the subsequent session was compared, until a significant difference was identified. Given the descriptive nature of this analysis, an uncorrected α value was used.

For the trained (middle) finger, significant improvement was observed immediately after the first training session [testing *session 2*: *t*(11) = 2.51, *P* = 0.029, *d* = 0.72]. For the adjacent index finger, the threshold became significant on the following testing session, conducted on the next day before the 2nd training session [testing *session 3*: *t*(11) = 3.92, *P* = 0.002, *d* = 1.13]. This indicates that the learning in the adjacent index finger “lagged” behind the trained finger such that significant gains were only apparent following the first consolidation period. Conversely, the adjacent ring finger's threshold became significant only on the final (5th) session [*t*(11) = 2.46, *P* = 0.032, *d* = 0.71, as above]. This session also followed an overnight consolidation, after the second day of training (see Fig. 2*A*).

#### Addressing alternative explanations for differential transfer of learning.

We wanted to address the possibility that the difference in learning over time between the two adjacent fingers occurred simply because of a different capacity between these fingers to learn i.e., from exposure to the repeated testing sessions. This could occur, for example, due to peripheral differences, such as mechanoreceptor density and skin conformance, or cortical differences, such as the size of cortical territory devoted to representing each finger. To rebut this account, we compared the improvements in thresholds of these fingers in the control group (which were untrained but improved in tactile threshold due to repeated testing) and of the untrained hand in the trained group (the hand that did not undergo training but may have learned from transfer and repeated testing).

In the control group, a repeated-measures ANOVA with factors Finger (2 levels: index and ring), Hand (2 levels: right and left), and Session (5 levels: *sessions 1–5*) revealed a significant main effect of Session [*F*(4,32) = 6.45, *P* = 0.001, η_p_^2^ = 0.45] and no significant interactions with the Finger or Hand factor [all *P* > 0.452; see Fig. 2*C* and Table 4]. This indicates that changes in the thresholds were consistent for the index and ring fingers of both hands in the control group.

**Table 4.** Details of main effects and interactions of Finger, Session, and Hand for comparisons of the index and ring fingers alone in control and trained (untrained hand only) groups

Factor	Finger (2) × Hand (2) × Session (5): Control Group Only	Finger (2) × Session (5): Trained Group (Untrained Hand Only)
Finger	*F*(1,8) = 20.29	*F*(1,11) = 14.89
	***P* = 0.002** (η^2^_p_ = 0.71)	***P* = 0.003** (η^2^_p_ = 0.58)
Session	*F*(4,32) = 6.45	*F*(4,44) = 2.50
	***P* = 0.001** (η^2^_p_ = 0.44)	*P* = 0.056 (η^2^_p_ = 0.19)
Hand	*F*(1,8) = 1.53	
	*P* = 0.251 (η^2^_p_ = 0.16)	
Finger × Hand	*F*(1,8) = 0.53	
	*P* = 0.488 (η^2^_p_ = 0.06)	
Session × Hand	*F*(1,8) = 0.28	
	*P* = 0.613 (η^2^_p_ = 0.03)	
Finger × Session	*F*(4,32) = 0.86	*F*(4,44) = 1.36
	*P* = 0.863 (η^2^_p_ = 0.04)	*P* = 0.263 (η^2^_p_ = 0.11)
Finger × Session × Hand	*F*(4,32) = 0.51	
	*P* = 0.515 (η^2^_p_ = 0.09)	

Values are main effects and interactions not included in the text (see results). Significance at *P* < 0.05 is indicated by bold text.

Finally, a repeated-measures ANOVA was performed to ensure no divergence in thresholds over time in the untrained index and ring fingers of the trained group (i.e., other index and other ring). There was no significant interaction of Finger × Session [*F*(4,44) = 1.36, *P* = 0.263, η_p_^2^ = 0.11]. Thus, in the untrained hand of the trained group, similarly to the control group, there was no difference in the change in thresholds across sessions between the index and the ring finger (see Fig. 2*B* and Table 4).

## DISCUSSION

In this study, consistent with previous research, we demonstrate that improved tactile acuity resulting from perceptual training to one finger selectively transfers over time to untrained fingers (Harrar et al. 2014; Harris et al. 2001). We further extend these findings by showing that learning transfers differently to two fingers that are both physically and cortically adjacent to the trained finger. This divergence in the rate of transfer across fingers may not have been identified previously because studies of tactile perceptual learning typically involved prolonged training with only a single posttest session for untrained fingers (Harrar et al. 2014; Harris et al. 1999; Kaas et al. 2013) or trained/untrained testing sessions that were not conducted concurrently (Sathian and Zangaladze 1997), or because results were averaged across finger identities (Harrar et al. 2014; Harris et al. 2001).

### 

#### Mechanism of topographic tactile perceptual learning.

Currently, the precise level of perceptual learning within the cortical hierarchy is debated. Some theories propose learning in lower order sensory areas, e.g., through plastic changes in tuning properties of sensory neurons (Adab and Vogels 2011; Jehee et al. 2012; Schoups et al. 2001; Shibata et al. 2011). Others highlight the role of read-out tuning of lower order sensory areas by higher order areas (e.g., frontal or decision-making areas: Kahnt et al. 2011; Law and Gold 2008; Petrov et al. 2005; Zhang et al. 2010). Regardless of the precise locus of learning and common to all of the proposed mechanisms, transfer of tactile perceptual learning occurs as a function of topographic organization and as such reflects processing in topographic areas of the somatosensory system.

Transfer of tactile perceptual learning has been suggested to occur as a function of overlap in inter-finger representations (Harrar et al. 2014; Harris and Diamond 2000; Harris et al. 1999). Electrophysiological work with nonhuman primates has revealed the scope for inter-finger overlap varies massively across the different cytoarchitectonic division of SI: later areas (such as areas 1 and 2) contain many neurons with whole hand or multifinger representations (reviewed in Iwamura et al. 2002), whereas “lower order” areas 3a and 3b contain more neurons with narrowly tuned RFs (Iwamura et al. 1993; Thakur et al. 2012). However, when the center of the RF is considered, rather than its spatial extent, representation in SI (area 3b in particular) is more spatially distributed than might be assumed from topographical mapping studies (e.g., microcolumnar structure in SI: McKenna et al. 1982; Tommerdahl et al. 1993; reviewed in Tommerdahl et al. 2010). Given the documented differences in individualized finger movements in monkeys and humans (as highlighted in the Introduction) and the limitations in elucidating RF properties in humans, it is unfortunately not yet possible to speculate which the cytoarchitectonic division(s) would have the topography required to underpin the results reported in this study; however, such knowledge may soon be afforded in humans by functional magnetic resonance imaging (fMRI) protocols capable of mapping overlap between the fingers in primary and secondary somatosensory cortex (e.g., see Besle et al. 2014; Tamè et al. 2012).

The source of topography in tactile perceptual learning could also be attributed to subcortical areas in the somatosensory hierarchy also containing digit topographies (e.g., spinal cord and cuneate nucleus: Florence et al. 1989). Indeed, evidence suggests that the decomposition of tactile stimuli (such as used in the current study) into spatial patterns begins with mechanoreceptive afferents in the periphery (Bensmaia et al. 2008) and cuneate nucleus (Jörntell et al. 2014). Because plasticity in SI appears to be influenced by reorganization in the cuneate nucleus (Kambi et al. 2014), it is likely that subcortical processes are inherently linked to the patterns of learning transfer reported in the current study and thus cannot be considered independently.

#### Divergent learning transfer between two topographically related fingers.

Though the index and the ring finger typically differ in their initial tactile thresholds (Sathian and Zangaladze 1996; Vega-Bermudez and Johnson 2001), we have demonstrated they may still have a similar same basic capacity to improve in spatial acuity from repeated exposure to tactile stimuli. This was suggested by learning in the control group that did not vary across fingers. Furthermore, in a previous study, we demonstrated no difference in the capacity for leaning following intensive training (either directly or through learning transfer) between the index and middle fingers (Harrar et al. 2014), which have considerable peripheral and central differences (Duncan and Boynton 2007; Shoham and Grinvald 2001). Although we cannot directly rule out the possibility that divergent learning in the trained hand might be somewhat affected by a different capacity for learning between the fingers, together these findings argue against this possibility. Instead, we suggest that this pattern likely reflects differing levels of overlap between somatosensory finger representations resulting from tactile coactivation during action.

Previous research has demonstrated distinct cooperative use patterns for different pairs of fingers (Häger-Ross and Schieber 2000; Soechting and Flanders 1997) and suggested a link between the independence of a finger in natural action and characteristics of its representation in the primary motor cortex (Ingram et al. 2008). Given that patterns of coincident and dissociated stimulation have been consistently shown to result in respective integration and segregation of RFs encoding the differentially stimulated areas (Mogilner et al. 1993; Wang et al. 1995), we suggest that the same mechanism may hold for sensory finger representation. Although we did not actively manipulate use patterns in the current task (i.e., our task was purely sensory), the dissociated rates of learning through transfer between the ring and index fingers (adjacent to the trained finger) appear to reflect differences in the cortical interrelationship of these fingers as a result of habitual patterns of finger manipulation (“use topography”). Consistent with this, differences in the overlap of middle and ring finger sensory maps have recently been identified in SI using high-resolution fMRI (see “overlap ratio,” Table 4 in Besle et al. 2014) and using representational similarity of multivoxel patterns (Ejaz et al. 2015). It should be reiterated, however, that the link between finger use patterns, cortical finger representation, and transfer of perceptual learning suggested in this report is tentative and requires causal support from future research.

The transfer of tactile perceptual learning between homologous fingers has been previously documented (Harrar et al. 2014; Harris et al. 2001; Nagarajan et al. 1998; Sathian and Zangaladze 1997, 1998). Currently, there is a dearth of literature regarding the statistics of natural actions between homologous fingers. For this reason, we are unfortunately unable to speculate about the link between habitual action and contralateral learning transfer. We therefore focus on within-hand transfer patterns, where the literature is sufficiently well grounded to build upon.

#### Topographic changes underlying divergent transfer of tactile learning.

Given that overlapping sensory representation was previously suggested to facilitate transfer of tactile learning (Harrar et al. 2014; Harris and Diamond 2000; Harris et al. 1999, 2001), the delayed improvement of the adjacent ring finger compared with the almost immediate learning in the adjacent index finger requires some discussion. We suggest that this is due to the increased overlap between ring and middle finger representations, which could be expected to result in faster transfer of learning to the ring than to the index finger. However, as mentioned in the Introduction, the somewhat counterintuitive result may reflect competitive cortical magnification processes that occur during training. Repetitive stimulation, as induced in the present study during the training session, has previously been shown to lead to lateral shifts of the representational borders of the stimulated zone and a migration of the foci of RFs toward the stimulated zone (Detorakis and Rougier 2014; Jenkins et al. 1990; Recanzone et al. 1992a; Xerri et al. 1994; see Buonomano and Merzenich 1998 for review). These neural changes occur in tandem with perceptual improvements (Recanzone et al. 1992a, 1993). We suggest that the expanding representation of the trained finger might recruit cortical territory from the two adjacent fingers and, furthermore, that this might have different immediate effects due to the differences in baseline overlap stated above. Thus, although both adjacent fingers receive benefits from learning transfer, we propose that this benefit is offset by the loss of neural resources to the trained finger.

Following cortical magnification of the middle finger zone, the relatively minor loss of shared territory (for the adjacent index compared with the adjacent ring) combined with perceptual gains from learning transfer would result in a net gain for the adjacent index finger. In comparison, the adjacent ring finger's loss of shared territory (to the trained finger undergoing cortical expansion) would represent a considerably larger proportion of the ring finger's overall territory, resulting in a net plateau, or even a temporary loss in tactile acuity. Consistent with evidence showing the transient effect of cortical magnification with training (Lissek et al. 2009; Reed et al. 2011), in the later stages of the experiment the adjacent fingers would regain their territory, resulting in a delayed improvement in discrimination threshold of the ring finger, replicating topographic tactile perceptual learning (Harrar et al. 2014).

As an alternative explanation to cortical magnification, differential transfer effects from the trained middle finger to its adjacent fingers could be the result of diverse excitatory and inhibitory synaptic plasticity in horizontal intracortical connections (Hickmott and Merzenich 2002; Paullus and Hickmott 2011). Consistent stimulation has been found to produce divergent outcomes depending on whether the stimulated connection occurs within a functionally defined region (i.e., a continuous connection) or across a functional border (discontinuous). For example, long-term potentiation of inhibitory circuits has been demonstrated to have a greater effect on continuous than discontinuous connections in SI (Paullus and Hickmott 2011). Applied to our results, the middle and ring finger representational zones should have a greater number of continuous connections between them than the middle and index finger representations because of the differential use patterns previously described. Learning in the middle finger would then lead to increased inhibition of continuous circuits (comparatively more inhibition in the ring finger representation) with concurrent excitation of discontinuous circuits (greater facilitation for the index representation) causing immediate improvement of index finger thresholds and a delayed improvement for the ring finger (see Muret et al. 2014 for discussion of a similar mechanism for divergent learning across the human hand-face border; see also Haenzi et al. 2014 for detrimental somatosensory outcomes following cross-border transfer).

Finally, consistent with a higher order explanation of learning, the divergent learning pattern might be explained at the level of read-out. During training, overlapping inputs would need to be inhibited to selectively read out from the middle finger, improving the signal-to-noise ratio and improving discrimination for the middle finger (Law and Gold 2008, 2009). Since at baseline the middle finger is thought to have greater overlapping representation with the trained finger, this inhibitory effect would cause the greatest detriment to the ring finger than to the index finger, causing the pattern of results reported presently.

Although speculative, these potential mechanisms fit with research demonstrating that intensive training in the fingers can lead to negative sensory and motor outcomes. For example, in cases of focal dystonia, it is suggested that extreme schedules of sensory training/exposure lead to cortical magnification and reduced intracortical inhibition through the loss of inhibitory interneurons (Hallett 2011). This results in the desegregation of finger representations (Butterworth et al. 2003) and alterations in the number of neurons with enlarged, overlapping, or multifinger RFs and the subsequent pain and motor deficits (Byl et al. 1996, 1997). This process also has been suggested to underlie the reductions in tactile sensitivity associated with ageing (Kalisch et al. 2009). Our findings therefore provide indirect support for the potential detrimental effects of competitive relationships between finger representations in the human brain.

#### Conclusions.

We report a difference in how tactile perceptual learning on the middle finger transfers to adjacent fingers. We suggest that whereas physical proximity is known to be an important organizing principle in SI (body topography), patterns of everyday activity could modulate basic body topography to reflect these interrelationships (use topography), with both processes occurring as a function of cooperative use patterns. We believe that these findings will aid in the development of a more complete understanding of the organizing principles of the somatosensory cortex and the importance of habitual patterns of motor activity in shaping representations in the somatosensory system.

## GRANTS

This work was supported by a European Commission Marie Curie Intra-European Fellowship (to T. R. Makin), a Sir Henry Dale Fellowship jointly funded by the Wellcome Trust and the Royal Society Grant 104128/Z/14/Z (to T. R. Makin), and a University of Queensland Graduate School International Travel Award (to H. Dempsey-Jones). V. Harrar is supported by a Canadian Institutes of Health Research Banting Postdoctoral Fellowship.

## DISCLOSURES

No conflicts of interest, financial or otherwise, are declared by the authors.

## AUTHOR CONTRIBUTIONS

H.D.-J. and J.O. performed experiments; H.D.-J., V.H., J.O., and T.R.M. analyzed data; H.D.-J., V.H., and T.R.M. interpreted results of experiments; H.D.-J. and T.R.M. prepared figures; H.D.-J., V.H., and T.R.M. drafted manuscript; H.D.-J., V.H., J.O., H.J.-B., C.S., and T.R.M. edited and revised manuscript; H.D.-J., V.H., J.O., H.J.-B., C.S., and T.R.M. approved final version of manuscript; V.H., J.O., and T.R.M. conception and design of research.

## References

[B1] Adab HZ, Vogels R Practicing coarse orientation discrimination improves orientation signals in macaque cortical area v4. Curr Biol 21: 1661–1666, 2011. 21962714 10.1016/j.cub.2011.08.037

[B2] Ahissar M, Hochstein S Task difficulty and the specificity of perceptual learning. Nature 387: 401–406, 1997. 9163425 10.1038/387401a0

[B3] Allard T, Clark S, Jenkins W, Merzenich M Reorganization of somatosensory area 3b representations in adult owl monkeys after digital syndactyly. J Neurophysiol 66: 1048–1058, 1991. 1753275 10.1152/jn.1991.66.3.1048

[B4] Belić JJ, Faisal AA Decoding of human hand actions to handle missing limbs in neuroprosthetics. Front Comput Neurosci 9: 27, 2015. 25767447 10.3389/fncom.2015.00027 PMC4341559

[B5] Bensmaia SJ, Denchev PV, Dammann JF, Craig JC, Hsiao SS The representation of stimulus orientation in the early stages of somatosensory processing. J Neurosci 28: 776–786, 2008. 18199777 10.1523/JNEUROSCI.4162-07.2008 PMC6670339

[B6] Besle J, Sánchez-Panchuelo RM, Bowtell R, Francis S, Schluppeck D Event-related fMRI at 7T reveals overlapping cortical representations for adjacent fingertips in S1 of individual subjects. Hum Brain Mapp 35: 2027–2043, 2014. 24014446 10.1002/hbm.22310 PMC4216413

[B7] Bleyenheuft Y, Thonnard JL Tactile spatial resolution measured manually: a validation study. Somatosens Mot Res 24: 111–114, 2007. 17853056 10.1080/08990220701496639

[B8] Buonomano DV, Merzenich MM Cortical plasticity: from synapses to maps. Annu Rev Neurosci 21: 149–186, 1998. 9530495 10.1146/annurev.neuro.21.1.149

[B9] Butterworth S, Francis S, Kelly E, McGlone F, Bowtell R, Sawle GV Abnormal cortical sensory activation in dystonia: an fMRI study. Mov Disord 18: 673–682, 2003. 12784271 10.1002/mds.10416

[B10] Byl NN, Merzenich MM, Cheung S, Bedenbaugh P, Nagarajan SS, Jenkins WM A primate model for studying focal dystonia and repetitive strain injury: effects on the primary somatosensory cortex. Phys Ther 77: 269–284, 1997. 9062569 10.1093/ptj/77.3.269

[B11] Byl NN, Merzenich MM, Jenkins WM A primate genesis model of focal dystonia and repetitive strain injury I. Learning-induced dedifferentiation of the representation of the hand in the primary somatosensory cortex in adult monkeys. Neurology 47: 508–520, 1996. 8757029 10.1212/wnl.47.2.508

[B12] Clark SA, Allard T, Jenkins WM, Merzenich MM Receptive fields in the body-surface map in adult cortex defined by temporally correlated inputs. Nature 332: 444–445, 1988. 3352741 10.1038/332444a0

[B13] Cousineau D Confidence intervals in within-subject designs: a simpler solution to Loftus and Masson's method. Tutor Quant Methods Psychol 1: 42–45, 2005.

[B14] Darainy M, Vahdat S, Ostry DJ Perceptual learning in sensorimotor adaptation. J Neurophysiol 110: 2152–2162, 2013. 23966671 10.1152/jn.00439.2013 PMC4073967

[B15] Detorakis GI, Rougier NP Structure of receptive fields in a computational model of area 3b of primary sensory cortex. Front Comput Neurosci 8: 76, 2014. 25120461 10.3389/fncom.2014.00076 PMC4112916

[B16] Duncan RO, Boynton GM Tactile hyperacuity thresholds correlate with finger maps in primary somatosensory cortex (S1). Cereb Cortex 17: 2878–2891, 2007. 17372277 10.1093/cercor/bhm015

[B17] Ejaz N, Hamada M, Diedrichsen J Hand use predicts the structure of representations in sensorimotor cortex. Nat Neurosci 18: 1034–1040, 2015. 26030847 10.1038/nn.4038

[B18] Flanders M Functional somatotopy in sensorimotor cortex. Neuroreport 16: 313–316, 2005. 15729128 10.1097/00001756-200503150-00001

[B19] Florence S, Wall J, Kaas J Somatotopic organization of inputs from the hand to the spinal gray and cuneate nucleus of monkeys with observations on the cuneate nucleus of humans. J Comp Neurol 286: 48–70, 1989. 2475533 10.1002/cne.902860104

[B20] Gibson EJ Principles of Perceptual Learning and Development. East Norwalk, CT: Appleton-Century-Crofts, 1969.

[B21] Haenzi S, Stefanics G, Lanaras T, Calcagni M, Ghosh A Botulinum toxin-A dose dependent perceptual loss on the hand after its cosmetic use on the face. Cortex 63: 118, 2014. 25282050 10.1016/j.cortex.2014.08.019

[B22] Häger-Ross C, Schieber MH Quantifying the independence of human finger movements: comparisons of digits, hands, and movement frequencies. J Neurosci 20: 8542–8550, 2000. 11069962 10.1523/JNEUROSCI.20-22-08542.2000 PMC6773164

[B23] Hallett M Neurophysiology of dystonia: the role of inhibition. Neurobiol Dis 42: 177–184, 2011. 20817092 10.1016/j.nbd.2010.08.025 PMC3016461

[B24] Harrar V, Spence C, Makin TR Topographic generalization of tactile perceptual learning. J Exp Psychol Hum Percept Perform 40: 15–23, 2014. 23855526 10.1037/a0033200

[B25] Harris JA, Diamond ME Ipsilateral and contralateral transfer of tactile learning. Neuroreport 11: 263–266, 2000. 10674467 10.1097/00001756-200002070-00008

[B26] Harris JA, Harris IM, Diamond ME The topography of tactile learning in humans. J Neurosci 21: 1056–1061, 2001. 11157091 10.1523/JNEUROSCI.21-03-01056.2001 PMC6762328

[B27] Harris JA, Petersen RS, Diamond ME Distribution of tactile learning and its neural basis. Proc Natl Acad Sci USA 96: 7587–7591, 1999. 10377459 10.1073/pnas.96.13.7587 PMC22130

[B28] Hickmott PW, Merzenich MM Local circuit properties underlying cortical reorganization. J Neurophysiol 88: 1288–1301, 2002. 12205150 10.1152/jn.00994.2001

[B29] Ingram JN, Kording KP, Howard IS, Wolpert DM The statistics of natural hand movements. Exp Brain Res 188: 223–236, 2008. 18369608 10.1007/s00221-008-1355-3 PMC2636901

[B30] Iwamura Y, Tanaka M, Iriki A, Taoka M, Toda T Processing of tactile and kinesthetic signals from bilateral sides of the body in the postcentral gyrus of awake monkeys. Behav Brain Res 135: 185–190, 2002. 12356449 10.1016/s0166-4328(02)00164-x

[B31] Iwamura Y, Tanaka M, Sakamoto M, Hikosaka O Rostrocaudal gradients in the neuronal receptive field complexity in the finger region of the alert monkey's postcentral gyrus. Exp Brain Res 92: 360–368, 1993. 8454001 10.1007/BF00229023

[B32] Jehee JF, Ling S, Swisher JD, van Bergen RS, Tong F Perceptual learning selectively refines orientation representations in early visual cortex. J Neurosci 32: 16747–16753, 2012. 23175828 10.1523/JNEUROSCI.6112-11.2012 PMC3575550

[B33] Jenkins WM, Merzenich MM, Ochs MT, Allard T, Guic-Robles E Functional reorganization of primary somatosensory cortex in adult owl monkeys after behaviorally controlled tactile stimulation. J Neurophysiol 63: 82–104, 1990. 2299388 10.1152/jn.1990.63.1.82

[B34] Johnson KO, Phillips JR Tactile spatial resolution. I. Two-point discrimination, gap detection, grating resolution, and letter recognition. J Neurophysiol 46: 1177–1192, 1981. 7320742 10.1152/jn.1981.46.6.1177

[B35] Jörntell H, Bengtsson F, Geborek P, Spanne A, Terekhov AV, Hayward V Segregation of tactile input features in neurons of the cuneate nucleus. Neuron 83: 1444–1452, 2014. 25175880 10.1016/j.neuron.2014.07.038 PMC4175181

[B36] Kaas AL, van de Ven V, Reithler J, Goebel R Tactile perceptual learning: learning curves and transfer to the contralateral finger. Exp Brain Res 224: 477–488, 2013. 23161157 10.1007/s00221-012-3329-8

[B37] Kaas JH, Nelson RJ, Sur M, Lin CS, Merzenich MM Multiple representations of the body within the primary somatosensory cortex of primates. Science 204: 521–523, 1979. 107591 10.1126/science.107591

[B38] Kahnt T, Grueschow M, Speck O, Haynes JD Perceptual learning and decision-making in human medial frontal cortex. Neuron 70: 549–559, 2011. 21555079 10.1016/j.neuron.2011.02.054

[B39] Kalisch T, Ragert P, Schwenkreis P, Dinse HR, Tegenthoff M Impaired tactile acuity in old age is accompanied by enlarged hand representations in somatosensory cortex. Cereb Cortex 19: 1530–1538, 2009. 19008462 10.1093/cercor/bhn190

[B40] Kambi N, Halder P, Rajan R, Arora V, Chand P, Arora M, Jain N Large-scale reorganization of the somatosensory cortex following spinal cord injuries is due to brainstem plasticity. Nat Commun 5: 3602, 2014. 24710038 10.1038/ncomms4602

[B41] Lang CE, Schieber MH Human finger independence: limitations due to passive mechanical coupling versus active neuromuscular control. J Neurophysiol 92: 2802–2810, 2004. 15212429 10.1152/jn.00480.2004

[B42] Law CT, Gold JI Neural correlates of perceptual learning in a sensory-motor, but not a sensory, cortical area. Nat Neurosci 11: 505–513, 2008. 18327253 10.1038/nn2070 PMC2424192

[B43] Law CT, Gold JI Reinforcement learning can account for associative and perceptual learning on a visual-decision task. Nat Neurosci 12: 655–663, 2009. 19377473 10.1038/nn.2304 PMC2674144

[B44] Lederman SJ, Klatzky RL Extracting object properties through haptic exploration. Acta Psychol (Amst) 84: 29–40, 1993. 8237454 10.1016/0001-6918(93)90070-8

[B45] Lissek S, Wilimzig C, Stude P, Pleger B, Kalisch T, Maier C, Peters SA, Nicolas V, Tegenthoff M, Dinse HR Immobilization impairs tactile perception and shrinks somatosensory cortical maps. Curr Biol 19: 837–842, 2009. 19398335 10.1016/j.cub.2009.03.065

[B46] McKenna T, Whitsel B, Dreyer D Anterior parietal cortical topographic organization in macaque monkey: a reevaluation. J Neurophysiol 48: 289–317, 1982. 7119852 10.1152/jn.1982.48.2.289

[B47] Mogilner A, Grossman J, Ribary U, Joliot M, Volkmann J, Rapaport D, Beasley RW, Llinas RR Somatosensory cortical plasticity in adult humans revealed by magnetoencephalography. Proc Natl Acad Sci USA 90: 3593–3597, 1993. 8386377 10.1073/pnas.90.8.3593 PMC46347

[B48] Morey RD Confidence intervals from normalized data: a correction to Cousineau (2005). Reason 4: 61–64, 2008.

[B49] Muret D, Dinse HR, Macchione S, Urquizar C, Farnè A, Reilly KT Touch improvement at the hand transfers to the face. Curr Biol 24: R736–R737, 2014. 25137581 10.1016/j.cub.2014.07.021

[B50] Nagarajan SS, Blake DT, Wright BA, Byl N, Merzenich MM Practice-related improvements in somatosensory interval discrimination are temporally specific but generalize across skin location, hemisphere, and modality. J Neurosci 18: 1559–1570, 1998. 9454861 10.1523/JNEUROSCI.18-04-01559.1998 PMC6792718

[B51] Overduin SA, d'Avella A, Carmena JM, Bizzi E Microstimulation activates a handful of muscle synergies. Neuron 76: 1071–1077, 2012. 23259944 10.1016/j.neuron.2012.10.018 PMC3547640

[B52] Overduin SA, d'Avella A, Carmena JM, Bizzi E Muscle synergies evoked by microstimulation are preferentially encoded during behavior. Front Comput Neurosci 8: 20, 2014. 24634652 10.3389/fncom.2014.00020 PMC3942675

[B53] Paullus JR, Hickmott PW Diverse excitatory and inhibitory synaptic plasticity outcomes in complex horizontal circuits near a functional border of adult neocortex. Brain Res 1416: 10–25, 2011. 21890112 10.1016/j.brainres.2011.07.062

[B54] Petrov AA, Dosher BA, Lu ZL The dynamics of perceptual learning: an incremental reweighting model. Psychol Rev 112: 715, 2005. 16262466 10.1037/0033-295X.112.4.715

[B55] Prins N, Kingdom FA Palamedes: Matlab routines for analyzing psychophysical data (Online). http://www.palamedestoolbox.org, 2009.

[B56] Recanzone GH, Schreiner CE, Merzenich MM Plasticity in the frequency representation of primary auditory cortex following discrimination training in adult owl monkeys. J Neurosci 13: 87–103, 1993. 8423485 10.1523/JNEUROSCI.13-01-00087.1993 PMC6576321

[B57] Recanzone GH, Merzenich MM, Jenkins WM, Grajski KA, Dinse HR Topographic reorganization of the hand representation in cortical area 3b owl monkeys trained in a frequency-discrimination task. J Neurophysiol 67: 1031–1056, 1992a. 1597696 10.1152/jn.1992.67.5.1031

[B58] Recanzone GH, Merzenich MM, Schreiner CE Changes in the distributed temporal response properties of SI cortical neurons reflect improvements in performance on a temporally based tactile discrimination task. J Neurophysiol 67: 1071–1091, 1992b. 1597698 10.1152/jn.1992.67.5.1071

[B59] Reed A, Riley J, Carraway R, Carrasco A, Perez C, Jakkamsetti V, Kilgard MP Cortical map plasticity improves learning but is not necessary for improved performance. Neuron 70: 121–131, 2011. 21482361 10.1016/j.neuron.2011.02.038

[B60] Reilly KT, Hammond GR Independence of force production by digits of the human hand. Neurosci Lett 290: 53–56, 2000. 10925173 10.1016/s0304-3940(00)01328-8

[B61] Reilly KT, Schieber MH Incomplete functional subdivision of the human multitendoned finger muscle flexor digitorum profundus: an electromyographic study. J Neurophysiol 90: 2560–2570, 2003. 12815024 10.1152/jn.00287.2003

[B62] Santello M, Flanders M, Soechting JF Postural hand synergies for tool use. J Neurosci 18: 10105–10115, 1998. 9822764 10.1523/JNEUROSCI.18-23-10105.1998 PMC6793309

[B63] Sathian K, Zangaladze A Perceptual learning in tactile hyperacuity: complete intermanual transfer but limited retention. Exp Brain Res 118: 131–134, 1998. 9547071 10.1007/s002210050263

[B64] Sathian K, Zangaladze A Tactile learning is task specific but transfers between fingers. Percept Psychophys 59: 119–128, 1997. 9038414 10.3758/bf03206854

[B65] Sathian K, Zangaladze A Tactile spatial acuity at the human fingertip and lip: bilateral symmetry and interdigit variability. Neurology 46: 1464–1466, 1996. 8628503 10.1212/wnl.46.5.1464

[B66] Schieber MH Individuated finger movements of rhesus monkeys: a means of quantifying the independence of the digits. J Neurophysiol 65: 1381–1391, 1991. 1875247 10.1152/jn.1991.65.6.1381

[B67] Schoups A, Vogels R, Qian N, Orban G Practising orientation identification improves orientation coding in V1 neurons. Nature 412: 549–553, 2001. 11484056 10.1038/35087601

[B68] Seitz AR, Dinse HR A common framework for perceptual learning. Curr Opin Neurobiol 17: 148–153, 2007. 17317151 10.1016/j.conb.2007.02.004

[B69] Shibata K, Watanabe T, Sasaki Y, Kawato M Perceptual learning incepted by decoded fMRI neurofeedback without stimulus presentation. Science 334: 1413–1415, 2011. 22158821 10.1126/science.1212003 PMC3297423

[B70] Shoham D, Grinvald A The cortical representation of the hand in macaque and human area S-I: high resolution optical imaging. J Neurosci 21: 6820–6835, 2001. 11517270 10.1523/JNEUROSCI.21-17-06820.2001 PMC6763104

[B71] Soechting J, Flanders M Flexibility and repeatability of finger movements during typing: analysis of multiple degrees of freedom. J Comput Neurosci 4: 29–46, 1997. 9046450 10.1023/a:1008812426305

[B72] Sterr A, Müller MM, Elbert T, Rockstroh B, Pantev C, Taub E Perceptual correlates of changes in cortical representation of fingers in blind multifinger Braille readers. J Neurosci 18: 4417–4423, 1998. 9592118 10.1523/JNEUROSCI.18-11-04417.1998 PMC6792812

[B73] Tamè L, Braun C, Lingnau A, Schwarzbach J, Demarchi G, Hegner YL, Farnè A, Pavani F The contribution of primary and secondary somatosensory cortices to the representation of body parts and body sides: an fMRI adaptation study. J Cogn Neurosci 24: 2306–2320, 2012. 22849401 10.1162/jocn_a_00272

[B74] Thakur PH, Fitzgerald PJ, Hsiao SS Second-order receptive fields reveal multidigit interactions in area 3b of the macaque monkey. J Neurophysiol 108: 243–262, 2012. 22457468 10.1152/jn.01022.2010 PMC3434610

[B75] Tommerdahl M, Favorov O, Whitsel B, Nakhle B, Gonchar Y Minicolumnar activation patterns in cat and monkey SI cortex. Cereb Cortex 3: 399–411, 1993. 8260808 10.1093/cercor/3.5.399

[B76] Tommerdahl M, Favorov OV, Whitsel BL Dynamic representations of the somatosensory cortex. Neurosci Biobehav Rev 34: 160–170, 2010. 19732790 10.1016/j.neubiorev.2009.08.009

[B77] Tresch MC, Cheung VC, d'Avella A Matrix factorization algorithms for the identification of muscle synergies: evaluation on simulated and experimental data sets. J Neurophysiol 95: 2199–2212, 2006. 16394079 10.1152/jn.00222.2005

[B78] Van Boven RW, Johnson KO The limit of tactile spatial resolution in humans: grating orientation discrimination at the lip, tongue, and finger. Neurology 44: 2361–2366, 1994. 7991127 10.1212/wnl.44.12.2361

[B79] Vega-Bermudez F, Johnson KO Spatial acuity after digit amputation. Brain 125: 1256–1264, 2002. 12023314 10.1093/brain/awf129

[B80] Vega-Bermudez F, Johnson KO Differences in spatial acuity between digits. Neurology 56: 1389–1391, 2001. 11376194 10.1212/wnl.56.10.1389

[B81] Volkman A Über den Einfluss der Übung. Ber Verh Sächs Wiss Leipzig Math-Phys Kl 10: 38–69, 1858.

[B82] Wang X, Merzenich MM, Sameshima K, Jenkins WM Remodelling of hand representation in adult cortex determined by timing of tactile stimulation. Nature 378: 71–75, 1995. 7477291 10.1038/378071a0

[B83] Weiss EJ, Flanders M Muscular and postural synergies of the human hand. J Neurophysiol 92: 523–535, 2004. 14973321 10.1152/jn.01265.2003

[B84] Xerri C, Stern JM, Merzenich MM Alterations of the cortical representation of the rat ventrum induced by nursing behavior. J Neurosci 14: 1710–1721, 1994. 8126565 10.1523/JNEUROSCI.14-03-01710.1994 PMC6577528

[B85] Zatsiorsky VM, Li ZM, Latash ML Coordinated force production in multi-finger tasks: finger interaction and neural network modeling. Biol Cybern 79: 139–150, 1998. 9791934 10.1007/s004220050466

[B86] Zhang JY, Zhang GL, Xiao LQ, Klein SA, Levi DM, Yu C Rule-based learning explains visual perceptual learning and its specificity and transfer. J Neurosci 30: 12323–12328, 2010. 20844128 10.1523/JNEUROSCI.0704-10.2010 PMC3842491

